# Sulodexide improves vascular permeability via glycocalyx remodelling in endothelial cells during sepsis

**DOI:** 10.3389/fimmu.2023.1172892

**Published:** 2023-08-08

**Authors:** Jiayun Ying, Caiyan Zhang, Yaodong Wang, Tingyan Liu, Zhenhao Yu, Kexin Wang, Weiming Chen, Yufeng Zhou, Guoping Lu

**Affiliations:** ^1^ Department of Critical Care Medicine, Children’s Hospital of Fudan University, Shanghai, China; ^2^ Institute of Pediatrics, Children’s Hospital of Fudan University, National Children’s Medical Center, and the Shanghai Key Laboratory of Medical Epigenetics, International Co-laboratory of Medical Epigenetics and Metabolism, Ministry of Science and Technology, Institutes of Biomedical Sciences, Fudan University, Shanghai, China; ^3^ National Health Commission (NHC) Key Laboratory of Neonatal Diseases, Fudan University, Shanghai, China; ^4^ State-level Reginal Children’s Medical Center, Children’s Hospital Of Fudan University at Xiamen (Xiamen Children’s Hospital), Fujian Provincial Key Laboratory of Neonatal Diseases, Fujian, China

**Keywords:** sepsis, sulodexide, glycocalyx remodeling, endothelium barrier function, Syndecan-1

## Abstract

**Background:**

Degradation of the endothelial glycocalyx is critical for sepsis-associated lung injury and pulmonary vascular permeability. We investigated whether sulodexide, a precursor for the synthesis of glycosaminoglycans, plays a biological role in glycocalyx remodeling and improves endothelial barrier dysfunction in sepsis.

**Methods:**

The number of children with septic shock that were admitted to the PICU at Children’s Hospital of Fudan University who enrolled in the study was 28. On days one and three after enrollment, venous blood samples were collected, and heparan sulfate, and syndecan-1 (SDC1) were assayed in the plasma. We established a cell model of glycocalyx shedding by heparinase III and induced sepsis in a mouse model via lipopolysaccharide (LPS) injection and cecal ligation and puncture (CLP). Sulodexide was administrated to prevent endothelial glycocalyx damage. Endothelial barrier function and expression of endothelial-related proteins were determined using permeability, western blot and immunofluorescent staining. The survival rate, histopathology evaluation of lungs and wet-to-dry lung weight ratio were also evaluated.

**Results:**

We found that circulating SDC1 levels were persistently upregulated in the non-alive group on days 1 and 3 and were positively correlated with IL-6 levels. Receiver operating characteristic curve analysis showed that SDC1 could distinguish patients with mortality. We showed that SDC1-shedding caused endothelial permeability in the presence of heparinase III and sepsis conditions. Mechanistically, sulodexide (30 LSU/mL) administration markedly inhibited SDC1 shedding and prevented endothelial permeability with zonula occludens-1 (ZO-1) upregulation via NF-κB/ZO-1 pathway. In mice with LPS and CLP-induced sepsis, sulodexide (40 mg/kg) administration decreased the plasma levels of SDC1 and increased survival rate. Additionally, sulodexide alleviated lung injury and restored endothelial glycocalyx damage.

**Conlusions:**

In conclusion, our data suggest that SDC1 predicts prognosis in children with septic shock and sulodexide may have therapeutic potential for the treatment of sepsis-associated endothelial dysfunction.

## Introduction

Sepsis is an immune dysregulation caused by an infection that results in organ dysfunction (1). It is one of the main causes of death in critically ill children. A recent study showed that sepsis-related deaths account for 19.7% of global deaths (2).

Vascular endothelial cells have an important role in regulating vascular smooth muscle tone, vessel barrier formation, systemic and local inflammation, and coagulation and thus play a central role in sepsis pathogenesis (3). The glycocalyx is a matrix coating of the luminal surface of vascular endothelial cells and is largely comprised of proteoglycans with attached or intercalated glycosaminoglycans (4, 5). Damage to the glycocalyx leads to endothelial dysfunction in sepsis, thereby increasing vascular permeability (6). Enzymatic damage to the glycocalyx reduces the expression of two endothelial cell proteins integral in maintaining vascular barrier function: zonula occludens-1 (ZO-1), present in tight junctions, and VE-cadherin integral to adherens junctions integrity (7). Syndecan-1 (SDC1), a heparan sulfate proteoglycan of the endothelial glycocalyx, affects the interaction of extracellular matrix components and soluble ligands with the cell surface. Prior work has demonstrated that SDC1 levels are elevated in adults with sepsis with higher SDC1 levels corresponding with greater mortality risk (8). Together, these data may suggest that in the setting of sepsis, shedding of SDC1 from the endothelial glycocalyx may increase endothelial permeability through a reduction in ZO-1/VE-cadherin expression.

Sulodexide is a composite of fast-mobility heparin and dermatan sulfate (9) with anti-inflammatory and glycocalyx-protective properties (10, 11). Its anti-inflammatory properties appear to lie in its ability to attenuate NF-kB activation as observed in human retinal endothelial cells (12). Moreover, its contribution to glycocalyx remodeling appears to attenuate endothelial monolayer permeability as observed in mouse models of sepsis (13). Though the precise mechanisms by which sulodexide contributes to glycocalyx restoration and endothelial cell signaling have not been fully characterized, this molecule serves as an attractive therapy to ameliorate sepsis-induced endotheliopathy.

Here, we report a previously unidentified role of SDC1 in mediating endothelial permeability during sepsis and its prognosis. This study revealed higher levels of plasma SDC1 in non-surviving septic shock children, compared with surviving children, and the levels were positively associated with IL-6. Notably, the administration of sulodexide promoted SDC1-remodeling and blockaded NF-κB pathway, resulting in improvement of endothelial permeability, along with ZO-1 upregulation. In addition, sulodexide administration attenuated SDC1 shedding and improved survival outcomes in murine sepsis. Our study suggests that sulodexide administration raises the prospect of preventing vascular leakage by remodeling the endothelial glycocalyx during sepsis.

## Methods

### Patient enrollment and sample collection

This prospective cohort study was registered with ClinicalTrials.gov (NCT03996720) and approved by the Children’s Hospital of Fudan University (Protocol No. 2018219). Pediatric patients (age 29 days to 18 years) were enrolled within 48 hours of admission to the pediatric intensive care unit (PICU) at Children’s Hospital of Fudan University for septic shock. The study period ran from January 2019 to December 2020. The diagnosis of septic shock was informed by the 2005 International Consensus Conference on Sepsis in Children Guidelines (14). The exclusion criteria were death within 24 h after entering the PICU, immunosuppressant use, or immunodeficiency. We collected and analyzed medical records, including demographic data and prognoses. Blood samples were collected in ethylenediaminetetraacetic acid (EDTA) tubes on the first and third day after inclusion and centrifuged at 400 × *g* for 10 min to separate plasma. The plasma was then frozen at -80°C for future analysis.

### Enzyme-linked immunosorbent assay

SDC1, heparan sulfate, and IL-6 were detected in human or mouse plasma using commercially available enzyme-linked immunosorbent assay (ELISA) kits (SDC1 human CD138, Abcam, UK; mouse SDC1, Jianglai, China; human heparan sulfate, Yaji, China; mouse heparan sulfate, Yaji, China; mouse IL-6, Kelu, China; human IL-6, Kelu, China). Plasma levels of SDC1, heparan sulfate, and IL-6 detected by ELISA were then calculated using four-parameter logistic curves generated by standards according to the manufacturer’s instructions.

### Animals

Specific pathogen-free (SPF) male C57BL/6J mice (3-4 weeks, 12-15 g) were obtained from JieSiJie Laboratory Animals (Shanghai, China). Appropriate temperature and humidity were controlled during a day-night cycle in the laboratory where the mice were housed. Food and water were easily obtained, and all animal experiments were conducted with ethical approval from the Animal Studies Committee of the Children’s Hospital of Fudan University (approval number: 2018219).

#### Endotoxemia model

The mice were randomly allocated to four groups (n = 5/experiment): LPS+SDX, LPS, SDX, and control. Within the groups, mice were injected intraperitoneally (*ip.*) with LPS (30 mg/kg body weight/mouse, Sigma, #L2630) and/or treated intragastrically (*ig.*) with sulodexide (40 mg/kg/mouse; Alfa Wassermann S.P.A.). Equal amounts of saline or sulodexide were injected into the control mice or mice in the SDX group. In the survival experiment, we recorded the mortality in each group three times a day for 120 h after LPS injection. For general anesthesia, 1% tribromoethanol was administered, and the mice were sacrificed 12 h later. Blood and lung samples were collected from the surviving mice.

#### CLP-induced polymicrobial sepsis model

We randomly grouped the mice into four (n = 5/experiment): control, CLP, SDX, and CLP+SDX. The CLP-induced sepsis model was developed based on previous literature (15). Briefly, after anesthesia with 1% tribromoethanol, laparotomy was performed. Cecum was ligated with 4-0 silk to 1 cm and punctured with a 22-gauge needle. Subsequently, a small mound of feces was squeezed from the hole after removing the needle. The peritoneum was sutured with a 6-0 silk suture, and the skin was intermittently sutured with a 4-0 silk suture. The same operation was performed on the control mice without ligation and perforation. The CLP mice were and/or intragastric (*ig.*) sulodexide (40 mg/kg). An equivalent volume of normal saline (NS) was injected into the control mice. After surgery, all the mice were subcutaneously resuscitated in 40 mL/kg saline. All the mice were sacrificed 24 h later. Retro-orbital blood and lung samples were collected from the surviving mice.

### Wet-to-dry lung weight ratio

The wet-to-dry (W/D) lung weight ratio indicated the degree of pulmonary edema. The left lungs were obtained and weighed. After the lungs were heated in oven at 60°C for 48 h, they were weighed again. The dry-to-wet weight ratio was then determined.

### Cell culture

Mouse microvascular endothelial cells (MLMECs) were obtained from the Core Technology Facility of Center for Excellence in Molecular Cell Science. Human umbilical vein endothelial cells (HUVECs) were provided by the Cell Bank of the Chinese Academy of Sciences Shanghai Branch (Shanghai, China). The passage number was from passage 3 (P3) to 8 (P8). The MLMECs/HUVECs were cultured in Dulbecco’s modified Eagle medium (DMEM) containing 10% fetal bovine serum (Gibco, MD, USA) and 1% streptomycin and penicillin (Hyclone). Cells were placed in an incubator at 37°C, 5% CO_2_, and 95% humidity.

### Cell treatment conditions

The MLMECs were allocated into four groups: (i) control group; cells were treated with serum-free medium for 2 h; (ii) heparinase III group; cells were treated with 15 mU/mL heparinase III (H8891, Sigam-Aldrich) for 2 h, 4 h, or 8 h, (iii) SDX group; cells were treated with 30 LSU/mL SDX for 2 h, and (iv) heparinase III+SDX group; cells were pretreated with 30 LSU/mL SDX for 2 h, then with 15 mU/mL heparinase III for 2 h, 4 h, or 8 h.

### Pulmonary histopathology evaluation

The left lungs of mice were removed and fixed in 4% paraformaldehyde. Subsequently, embedding, dewaxing, and hydration were performed, after which the sections were cut into slides, stained with hematoxylin and eosin, and observed under a microscope. The degree of lung injury was evaluated based on the degree of inflammatory cell infiltration and congestion of the lung tissue.

### Immunofluorescent staining of lung tissues, MLMECs, and HUVECs

Lung tissue sections were deparaffinized with xylene and dehydrated with ethanol. The antigen was retrieved before immunofluorescence, and MLMECs were grown on a Confocal Dish (Dianrui, Shanghai, China) with proper treatment. Cells were fixed with 4% paraformaldehyde and ruptured using 0.1% Triton X-100. After blocking with 5% donkey serum, cells were prepared for immunofluorescence. Both were incubated with goat anti-mouse primary antibody to SDC1 (1:100; af3190, R&D Systems). They were then incubated with secondary antibodies conjugated to Alexa Fluor-488 (1:100; A12398, Thermo Scientific). For immunocytochemistry, HUVECs were incubated with rabbit anti-human primary antibody to SDC1 (1:100; ab128936, Abcam). They were then incubated with secondary antibodies conjugated to Alexa Fluor-488 (1:100; ab150077, Abcam). For the detection of heparan sulfate, MLMECs/HUVECs were incubated with primary mouse IgM to F58-10E4 (1:100; 370255-S, Amsbio) and then with secondary antibodies conjugated to Alexa Fluor-488 (1:1000; A10680, Thermo Scientific). Cell nuclei were counterstained with 4,6′-diamidino-2-phenylindole (DAPI). After staining, the cells were observed under a confocal laser scanning microscope SP8 (Leica Microsystems, Wetzlar, Germany), and images were recorded with LAS AF Lite 2.6.0 (Leica Microsystems, Wetzlar, Germany).

### Western blot

MLMECs were lysed using RIPA buffer (Beyotime Biotechnology, Shanghai, China) containing protease and phosphatase inhibitors (Thermo Fisher Scientific, Carlsbad, CA, USA). Equal amount of protein (20 g per well) was added to a sodium dodecyl sulfate-polyacrylamide gel and separated by electrophoresis. The protein was transferred onto PVDF membranes (Millipore). Membranes were blocked with 5% skim milk in 0.1% Tween 20. Next, the membrane was incubated with primary antibody at 4°C. The membrane was then incubated with a horseradish peroxidase-conjugated secondary antibody. ECL system (Invitrogen) was used to detect signals. The following primary antibodies were used: ZO-1 (1:1000; 339100l, Invitrogen), VE-cadherin (1:1000; ab33168, Abcam), NF-κB/p65 (1:1000; 8242S, Cell Signaling Technology), NF-KB/p-p65 (1:1000; 3033S, Cell Signaling Technology), and β-actin (1:5000; 3700S, Cell Signaling Technology).

### RNA extraction and quantitative real-time PCR analysis

TRIzol reagent was used to extract total RNA from MLMECs. Reverse transcription was performed using a reverse transcription kit (Takara, Tokyo, Japan) according to the manufacturer’s instructions. RT-qPCR was performed using the SYBR Premix Ex Taq (Takara, Tokyo, Japan) on a LightCycler 480 II (Roche, Sweden). The evaluation of relative expression of mRNA was through the 2^−ΔΔCt^ method which normalized the expression of GAPDH. The primers used for RT-qPCR are listed in **Supplemental Table 1**.

### Evaluation of endothelium barrier permeability

MLMECs were cultured using the Transwell system (0.4 μm pore size polyester membrane inserts, Corning, Union City, CA, USA). Measurement of FD40 across the endothelium was used to evaluate endothelial barrier permeability (16). The MLMECs were treated with or without heparinase III (15 mU/mL) or SDX (30 LSU/mL). We added 0.1 mg/mL of FD40 to the upper inserts and an equal amount of serum-free medium was added to the lower compartments of the Transwell system for 60 min. Fluorescence across the upper inserts was measured at excitation and emission wavelengths of 490 and 520 nm, respectively.

### Statistical analyses

All experiments were carried out at least 3 times independently. After conducting a normality test, data was analyzed by the Student’s t-test or one-way ANOVA followed by Tukey’s analysis (for normally distributed data); and showed as the means ± SEM through SPSS v21.0 (SPSS Inc., Chicago, IL, USA). The Receiver Operating Characteristic (ROC) curve was used to determine the area under the curve (AUC) for SDC1 expression. GraphPad Prism 7.00 (GraphPad Software, La Jolla, CA, USA) and Adobe Photoshop CC 14.0 (Adobe, San Jose, CA, USA) were used for processing. p values less than 0.05 were considered significant.

## Results

### SDC1 is a predictor of prognosis in children with septic shock

**Figure 1:**
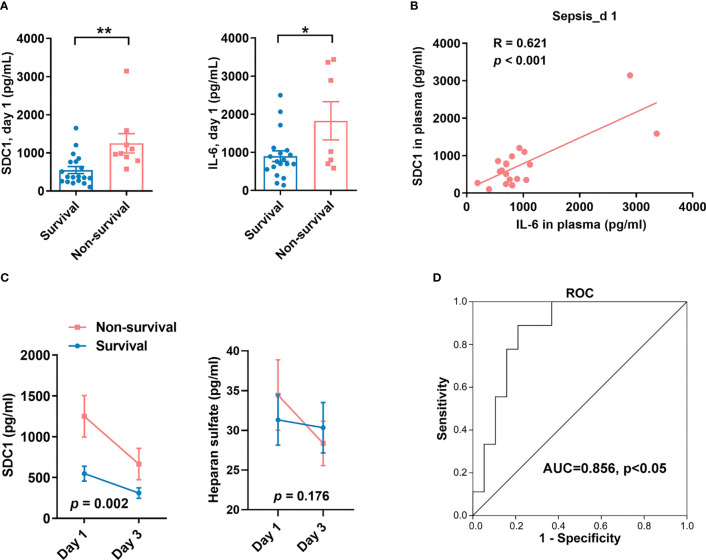
Syndecan-1 (SDC1) is a prognosis biomarker of children with septic shock. **(A)** ELISA to detect the levels of SDC1and IL-6 in plasma from 28 children with sepsis within 24 hours of PICU admission (day 1). **(B)** Correlation between levels of IL-6 and SDC1. **(C)** Changes of levels of SDC1 and heparan sulfate in plasma from day 1 to day 3. **(D)** ROC curve analysis of SDC1 expression on day 1 between survival group and non-survival group. Bars and error bars represent the mean ± SEM; *p < 0.05, **p < 0.01.

We studied 28 children with septic shock who were admitted to the PICU of Children’s Hospital of Fudan University. Of these patients, nine died and 19 survived 28 days after admission (**Supplemental Table 2**). We found that SDC1 levels in the non-survival group were significantly upregulated compared to those in the survival group with septic shock on the first (day 1) and third day (day 3). On day 1, the median SDC1 levels in patients in the survival and non-survival groups were 375 (IQR 258-769) ng/mL and 983 (IQR 843-1398) ng/mL, respectively (*p* = 0.002). IL-6 levels were also significantly upregulated in the non-survival groups (**Figure 1A**). However, there was no difference in the levels of heparan sulfate between the survival and non-survival groups on day 1 (**Figure S1A**). Higher SDC1 levels were associated with higher IL-6 levels (**Figure 1B**). However, on day 3, the levels of SDC1, unlike heparan sulfate, were still higher in patients in the non-survival group (median 573 (IQR 257-914) ng/mL) than those in the survival group (median 210 (IQR 124-499) ng/mL) (*p* = 0.05) (**Figure S1B**). The SDC1 levels decreased over time in the two groups compared to heparan sulfate levels (**Figure 1C**). The ROC curve showed that the accuracy of plasma SDC1 on day 1 for predicting mortality was 0.856 (**Figure 1D**). Taken together, these data suggest that SDC1 expression is a prognostic predictor in children with septic shock.

### Sulodexide decreased heparinase III-induced shedding of SDC1 in MLMECs

SDC1 is a member of a small family of transmembrane proteoglycans that are mainly expressed on the cell surface, and the main marker of endothelial glycocalyx degradation (17). To reduce the effect of endothelial cell inflammation on the glycocalyx stimulated by LPS, we used heparinase III as a specific hydrolytic agent of the glycocalyx to explore the effect of glycocalyx shedding on endothelial cells. Although heparinase III is not predicted to cause SDC1 shedding based upon its expected site of activity at heparan sulfate molecules, reducing the amount of heparan sulfate by addition of bacterial heparinase III can elevate SDC1 shedding dramatically (18–20). Sulodexide is a glycosaminoglycan consist of heparan sulfate and dermatan sulfate with anti-inflammatory property, which reduces the release of LPS-stimulated inflammatory mediators from macrophages (21). Heparin and heparin derivatives, as the main components of sulodexide, are believed to bind acute-phase and complement proteins, cytokines, and growth factors (22). In addition, sulodexide has numerous antiproteolytic effects via modulation of serine and metalloprotease enzymes, and matrix metalloproteinases, which are also involved in shedding of the glycocalyx (23).

**Figure 2:**
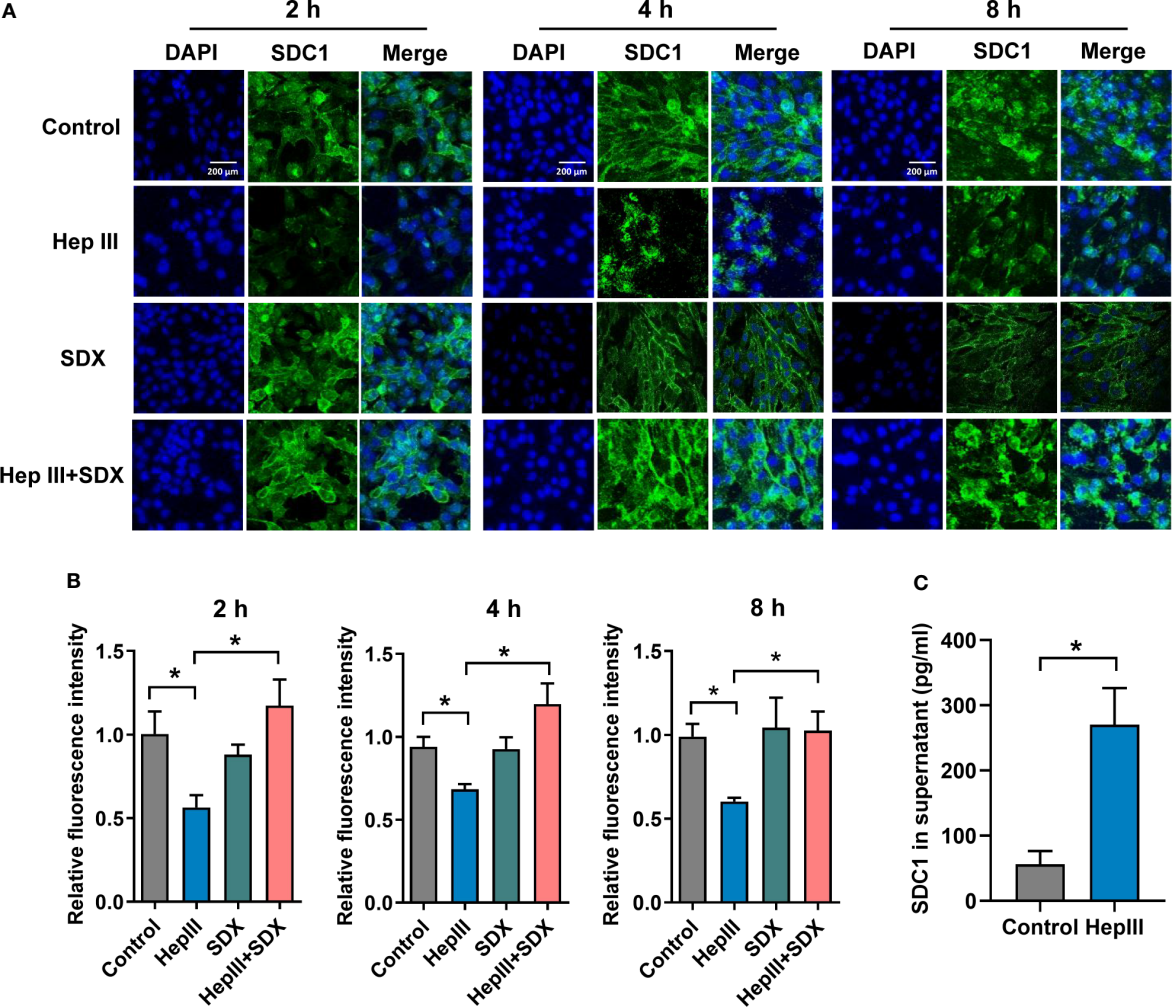
Sulodexide decreased heparinase III-induced shedding of SDC1 in MLMECs. **(A)** MLMECs were treated with 15 mU/mL Hep III for 2 h, 4 h, or 8 h. Cells in Hep III+SDX group were treated with 30 LSU/mL sulodexide for 2 h before. Cells in the SDX group were treated with sulodexide for 2 h, and then with PBS in the same volume. Representative image of immunofluorescence of SDC1 on MLMECs, scale bar = 200 μm. **(B)** Densitometry of immunofluorescence of SDC1 on MLMECs. **(C)** ELISA to detect the levels of SDC1 in MLMECs supernatant with treatment of Hep III for 2 h. Data were expressed as the means ± SEM; *p < 0.05.

To test the effect of sulodexide on the endothelial glycocalyx layer, we performed MLMECs and HUVECs culture conditions in presence of heparinase III. As expected, heparan sulfate was removed from the endothelium by heparinase III (**Figures S2A, B**). Notably, the administration of heparinase III also induced the shedding of SDC1 from the membrane of MLMECs, and the administration of supplemental sulodexide significantly presented a rescue at different times (**Figures 2A–C**). The same result was found in HUVECs (**Figures S2C, D**). Next, we assessed whether sulodexide increased SDC1 levels in MLMECs by upregulating the expression of the *SDC1* gene. Therefore, we measured *SDC1* mRNA expression under these conditions. However, we found no difference in the different treatment groups on *SDC1* mRNA expression (**Figure S2E**). Thus, these data showed that sulodexide could improve heparinase III-induced SDC1 expression, but not by increasing *SDC1* gene expression.

### Sulodexide prevented endothelial permeability through enhancing ZO-1 expression

**Figure 3:**
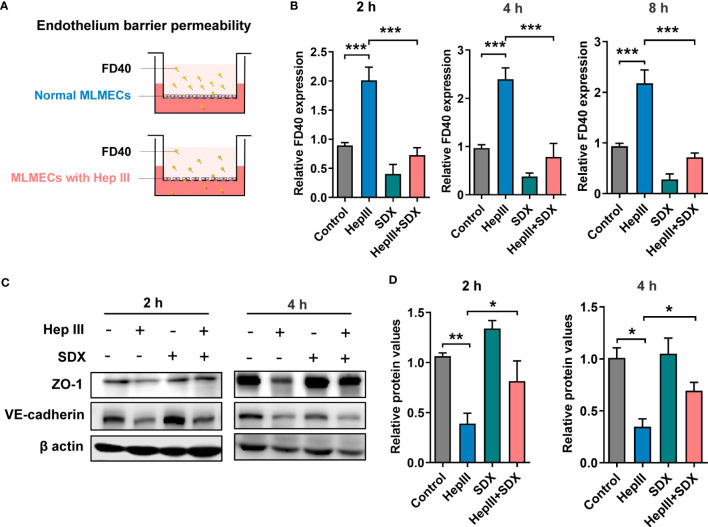
Sulodexide improved endothelial permeability resulting from glycocalyx shedding-induced ZO-1 disruption. **(A)** Pattern diagram for transwell model. MLMECs were treated with 15 mU/mL Hep III for 2 h, 4 h or 8 h, and/or 30 LSU/mL SDX for 2 h, respectively. **(B)** Relative fluorescence value of FD40 that passed through the inserts was assayed for 2 h, 4 h, or 8 h. **(C)** Levels of ZO-1 and VE-cadherin were quantified by western blot for 2 h or 4 h. **(D)** Statistical analysis of the levels of ZO-1. Data were expressed as means ± SEM; *p < 0.05, **p < 0.01, ***p < 0.001.

The effect of sulodexide on EC barrier function was assessed using a Transwell system (**Figure 3A**). We found that SDX decreased heparinase-III-induced endothelial permeability (**Figure 3B**). Sulodexide also prevented heparinase-induced endothelial cell permeability in HUVECs (**Figure S3A**). Interestingly, permeability improved in the SDX group compared to the control group. VE-cadherin and ZO-1 were important components that play crucial roles in the maintenance of EC barrier (24, 25). We aimed to determine whether endothelial barrier dysfunction due to SDC1 shedding is associated with ZO-1 and VE-cadherin. Notably, 2 h or 4 h after treatment with heparinase III, the levels of VE-cadherin and ZO-1 markedly decreased, as determined by western blot analysis (**Figures 3C, D**). Interestingly, the administration of supplemental sulodexide upregulated heparinase III-induced ZO-1 levels rather than VE-cadherin levels (**Figures 3C, D**; **S3B, C**). Altogether, these data suggest that sulodexide can prevent glycocalyx shedding-induced endothelial permeability by increasing ZO-1 expression.

### Sulodexide improved permeability via abolishing activation of NF-κB signaling

**Figure 4:**
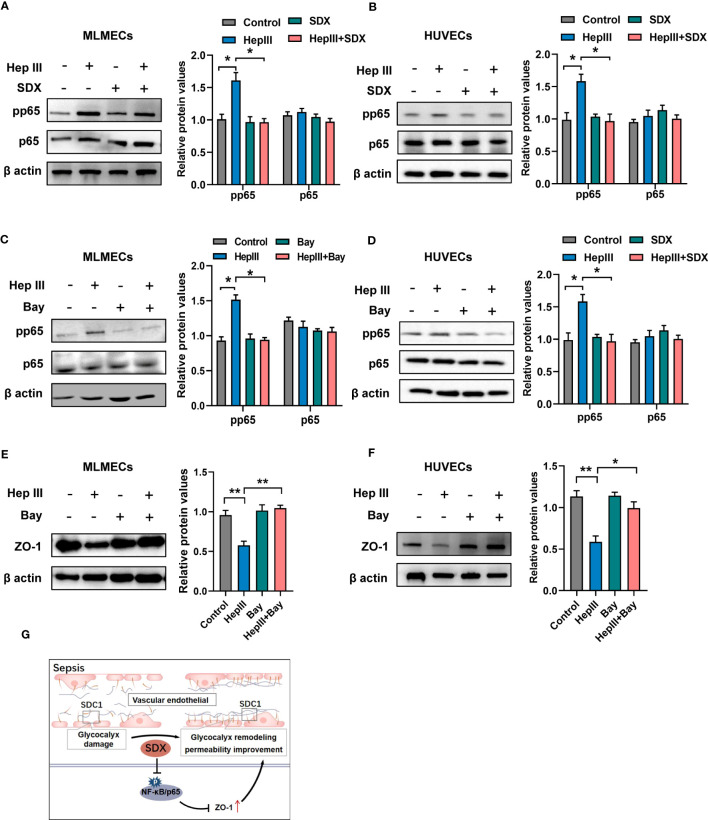
Sulodexide promoted permeability via abolishing activation of NF-κB signaling **(A, B)** MLMECs **(A)**/HUVECs **(B)** were treated with 15 mU/mL Hep III for 15/30 min. Cells in the Hep III+SDX group were pre-treated with 30 LSU/mL sulodexide for 2 h. Cells in the SDX group were treated with sulodexide for 2 h without Hep III. **(C, D)** MLMECs **(C)**/HUVECs **(D)** were treated with 40 μM/mL Bay 11- 7082 for 12 h. Cells were then treated with or without 15 mU/mL Hep III for 15/30 min. The levels of NF-κB/p-p65 and NF-κB/p65 were quantified by western blot. **(E, F)** MLMECs €/HUVECs **(F)** were treated with 40 μM/mL Bay 11- 7082 for 12 h. Cells were then treated with or without 15 mU/mL Hep III for 15/30 min. The levels of ZO-1 were quantified by western blot. **(G)** A schematic pattern. SDX protects vascular permeability via glycocalyx remodeling against sepsis *in vivo* and *in vitro*. Data were expressed as the means ± SEM; *p < 0.05, **p <0 .01.

Under various pathophysiological conditions, NF-κB plays an important role in inflammatory phenotypic changes as a transcription factor in endothelial cells (26). After glycocalyx disruption, shear stress leads to the upregulation of ICAM-1 protein expression and increased NF-κB activation (27). However, it is unclear whether the expression of ZO-1 is mediated by the NF-κB pathway when heparinase III degrades the glycocalyx. To address this, we evaluated the levels of NF-κB/p-p65 and NF-κB/p65 in total. Phosphorylated p65 levels markedly increased within 15/30 min of heparinase treatment and decreased with the addition of sulodexide in MLMECs/HUVECs (**Figures S4A, B**). With the administration of sulodexide, phosphorylated p65 decreased after heparinase treatment in MLMECs/HUVECs (**Figures 4A, B**). Similarly, the administration of Bay 11-7082, an inhibitor of NF-κB, markedly attenuated heparinase III-induced increases in phosphorylated p65 and increased the expression of ZO-1 (**Figures 4C-F**). Collectively, our results revealed that the activation of NF-κB signaling contributes to the expression of ZO-1 when heparinase III degrades the glycocalyx in MLMECs/HUVECs. Notably, sulodexide can promote the remodeling of glycocalyx and improve permeability by preventing NF-κB/ZO-1 signaling overactivation (**Figure 4G**).

### Inhibition of SDC1 shedding by sulodexide improved lung injury and prevented death in septic mice

**Figure 5:**
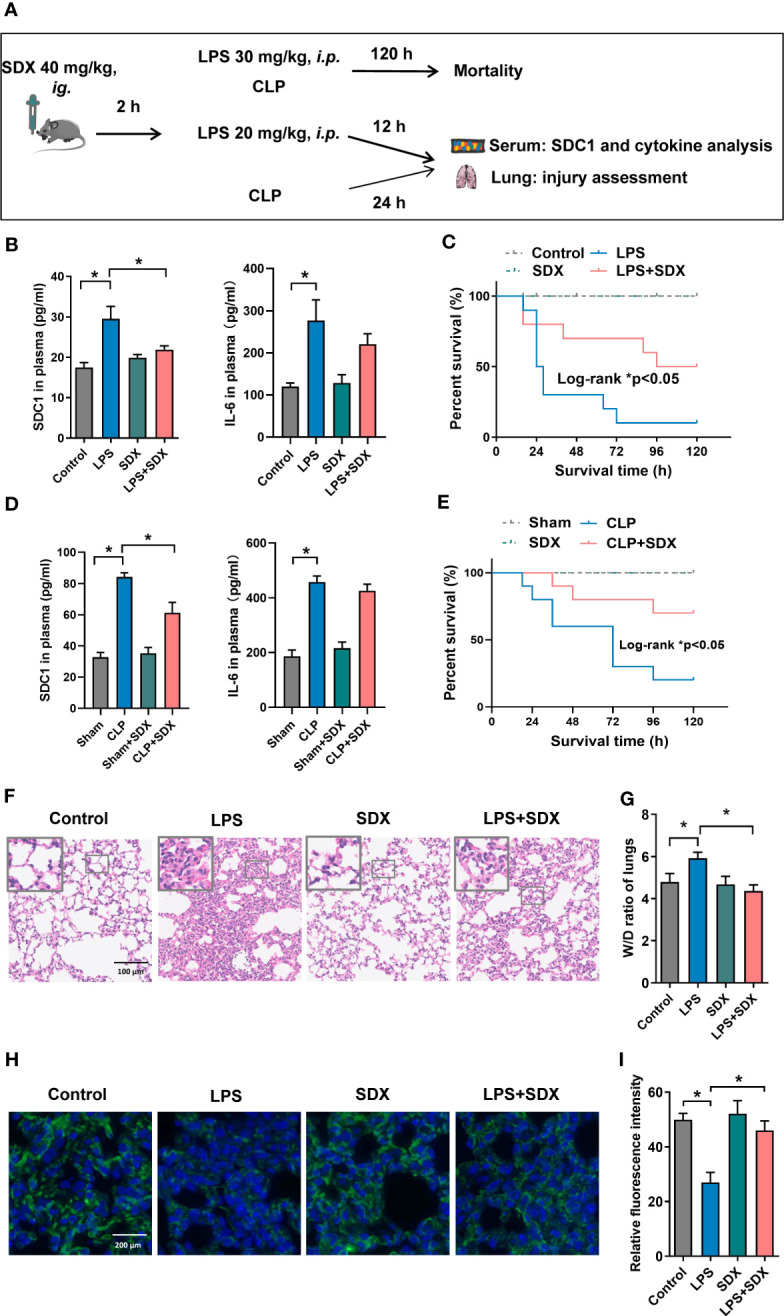
Inhibition of SDC1 shedding by Sulodexide improved lung injury and survival in septic mice. **(A)** Pattern diagram of the septic mouse model. Mice were pretreated with sulodexide (40 mg/kg) for 2 h before LPS injection or CLP procedure and those mice were observed for 12 h following LPS and 24 h following CLP before euthanasia. **(B, D)** ELISA to detect the plasma levels of SDC1 and IL-6 in the LPS (20 mg/kg)-challenged **(B)** and CLP-induced **(D)** septic model. **(C, E)** Survival curves after sulodexide administration to LPS (30 mg/kg)-challenged **(C)** and CLP-induced **(E)** mice, n=10/group/experiment. **(F)** Representative lung tissue sections stained with HE at ×100 **(D)** in LPS-induced sepsis model. **(G)** Lung tissue W/D weight ratio in LPS- induced sepsis model. **(H)** Representative immunofluorescence images of SDC1 in lungs, scale bar = 200 μm. **(I)** Densitometry of SDC1 immunofluorescence in lungs. Data are expressed as means ± SEM; *p < 0.05.

We established a sepsis model using CLP and LPS (**Figure 5A**). First, we measured SDC1 levels in the plasma of mice with sepsis. We found that SDC1 was upregulated in mice with sepsis. With the administration of sulodexide, the SDC1 level was downregulated, and the survival rate of mice with sepsis improved (**Figures 5B-E**). However, sulodexide treatment did not reduce IL-6 levels during sepsis (**Figures 5B, D**). Taken together, these data suggested that sulodexide restored the survival rate and decreased SDC1 expression in the plasma of mice with sepsis.

We evaluated the histology of the lungs in mice. Notably, in the LPS/CLP group, lung tissues were remarkably damaged, unlike those in the control group. Compared with the LPS/CLP group, there was a marked improvement in morphological features in the LPS+SDX/CLP+SDX group (**Figures 5F**, **S5A**). To determine pulmonary vascular permeability in mice, we measured the wet/dry (W/D) ratio of lung tissues. As expected, the LPS/CLP increase in W/D in the lung tissues were significantly inhibited by sulodexide (**Figures 5G**, **S5B**). Finally, we assessed glycocalyx damage by measuring SDC1 expression lung tissues fluorescent labeling of the lung tissue. In the sepsis model, SDC1 expression was lower than in the control group. Sulodexide helped preserve SDC1 expression in the lung tissues of the sepsis model (**Figures 5H, I**, **S5C**). Altogether, these data show that sulodexide prevented lung injury and sepsis-induced the shedding of endothelial glycocalyx in mice.

## Discussion

This study provides evidence of the protective effects of sulodexide on the glycocalyx induced by sepsis and heparinase III. First, we found that SDC1 levels in the plasma of children with septic shock were related to prognosis. *In vitro*, we observed that sulodexide inhibited the shedding of SDC1 and heparinase III-provoked hyperpermeability in MLMECs, and significantly restored the heparinase III-induced suppression of ZO-1, but not VE-cadherin. Furthermore, we found that the permeability improves via the NF-κB/ZO-1 pathway when heparinase III degrades glycocalyx. Third, sulodexide downregulated SDC1 levels *in vivo* and restored the survival rate of mice with sepsis. Additionally, sulodexide alleviated lung injury and restored endothelial glycocalyx damage.

Sepsis is a dysregulated response to an infection that affects all organs. The endothelium is an important component involved in the process of sepsis (28). Endothelial function can affect sepsis prognosis. The glycocalyx on the surface of the endothelium forms a part of the endothelial function. During sepsis, the glycocalyx may be shed by various mechanisms, such as metalloproteinases, heparanase, and hyaluronidases (29). SDC1 and heparan sulfate are the main components of glycocalyx (30). Previous studies have shown that SDC1 is a prognostic marker in patients with sepsis (31, 32). Our findings confirm that SDC1 is associated with mortality. However, in our study, the other two components were not related to the prognosis in children with septic shock. Thus, SDC1 may serve as a biomarker for the prognosis of children with sepsis.

In vascular homeostasis, during sepsis, heparanase causes the glycocalyx to shed (33). Heparanase can degrade heparan sulfate chains of the glycocalyx. Heparanase inhibition may have beneficial effects on endothelial function by blocking glycocalyx shedding and disruption (34). Doxycycline and heparin were used to inhibit glycocalyx degradation in previous studies (35, 36). Sulodexide is similar in composition to glycocalyx and has an anti-heparanase effect (37). In this study, treatment of MLMECs exposed to heparinase III with sulodexide was effective in preventing glycocalyx degradation, especially with SDC1. *In vivo*, sulodexide restored the survival rate and decreased plasma expression of SDC1 in septic mice. In addition, sulodexide alleviated lung injury and restored endothelial glycocalyx damage in mice with sepsis. Consistent with our data, sulodexide accelerated EG regeneration *in vitro* (13).

The traditional Starling’s principle is thought to regulate vascular permeability. Hydrostatic and oncotic forces inside and outside the vascular lumen determine filtration across the microvasculature. However, the glycocalyx, as the structural determinant of the oncotic gradient, is a part of the revised Starling equation (38, 39). Sulodexide can restore the shredded endothelial glycocalyx and prevent further degradation. A previous study showed that glycocalyx perturbations and increased vascular permeability were partially reversed following sulodexide administration in patients with type 2 diabetes (40). In our preliminary experiments, the significant increase in heparinase III-induced endothelial permeability was curtailed by sulodexide treatment. Correction of endothelial permeability is likely related to the structural and functional restoration of the glycocalyx. In addition, the restoration of cell-to-cell junctions can decrease endothelial permeability. In a previous study, upregulation of SDC1 significantly restored occludin and ZO-1 expression in sepsis (41). In this study, we found that sulodexide upregulated ZO-1 expression following heparinase III administration. However, VE-cadherin expression was not restored by the sulodexide treatment. Thus, sulodexide may contribute to the synthesis of the endothelial glycocalyx via an unclear mechanism.

It has been demonstrated that NF-κB is a transcription factor of inflammation in sepsis. Previous studies reported that when the glycocalyx was removed, the activity of NF-κB changed (27). Consistently, sulodexide protects human retinal endothelial cells from high-glucose damage via downstream NF-κB activity (12). However, MCTR1 and PCTR1 stabilized the glycocalyx via NF-κB pathway, thereby improving the endothelial barrier in sepsis (42, 43). In this study, we found that sulodexide also restored endothelial glycocalyx and improved permeability via the NF-κB/ZO-1 pathway. A previous study showed that SDC1-high-Exos or overexpression of SDC1 overexpression were significantly associated with endothelial function restoration (44).

There were several important limitations to this study. Heparinase III, which specifically hydrolyzes heparan sulfate and can directly lead to glycocalyx shedding, was preferred as a stimulus for *in vitro* glycocalyx investigation in our study. However, in the presence of heparinase III, SDC1 and heparan sulfate were both degraded. A method to specifically shed heparan sulfate or SDC1 *in vitro* is lacking. Moreover, SDC1 and heparan sulfate affect NF-κB activation in previous studies (44–46). In this study, we attempted to link heparinase III-induced SDC1 shedding to downstream upregulation of p65 activity and resultant loss of ZO-1 expression. However, avoiding the influence of heparan sulfate remains difficult. The investigation on SDC1 and heparan sulfate in NF-κB/ZO-1 signaling remains to be comprehensively established. Moreover, heparan sulfate fragments may promote NF-κB signaling in endothelial cells (47). Therefore, we cannot completely rule-out an increase in NF-κB signaling by shed heparan sulfate fragments released from the endothelial glycocalyx following heparinase III treatment. Finally, our *in vitro* experiments were performed under static conditions. Conditioning endothelial cells with shear stress impacts glycocalyx composition (48) and more closely recapitulates the *in vivo* environment. Therefore, further work is required to validate our findings in flow conditioned endothelial cells.

In summary, this study demonstrated a previously unknown role of SDC1 in prognosis for children with septic shock and promoting vascular permeability by inducing ZO-1 disruption mediated by NF-κB dependent signaling in ECs. Sulodexide administration may thus serve as a helpful treatment in sepsis by attenuating glycocalyx shedding and downstream EC signaling that promotes vascular leakage.

## Data availability statement

The raw data supporting the conclusions of this article will be made available by the authors, without undue reservation.

## Ethics statement

The studies involving humans were approved by the Ethics Board in the Children’s Hospital of Fudan University. The studies were conducted in accordance with the local legislation and institutional requirements. Written informed consent for participation in this study was provided by the participants’ legal guardians/next of kin. The animal study was approved by the Ethics Board in the Children’s Hospital of Fudan University. The study was conducted in accordance with the local legislation and institutional requirements.

## Author contributions

YZ and GL designed, supervised, and coordinated the overall research. JY and CZ developed the experimental protocol and analyzed the experiments. YW carried out additional work in response to comments from reviewers. TL, KW, and YW contributed to the development of the experimental protocols. JY and TL collected clinical samples. JY, CZ, WC, YZ. and GL wrote the manuscript. All authors contributed to the article and agreed to submit the manuscript.

## Supplementary material

Supplementary Figure 1 Glycocalyx as biomarkers to predict the prognosis of children with septic shock. **(A)** ELISA to detect the levels of heparan sulfate in plasma from children with sepsis on day 1. **(B)** Levels of SDC1 and heparan sulfate in plasma from septic children on day 3. Bars and error bars represent the mean ± SEM; **p* < 0.05; ns, no significance.

Supplementary Figure 2 Sulodexide decreased heparinase III-induced shedding of SDC1 in MLMECs/HUVECs. **(A)** MLMECs or HUVECs were treated with 15 mU/mL Hep III for 2 h. Representative images of immunofluorescence of heparan sulfate, scale bar = 200 μm. **(B)** ELISA to detect the levels of heparan sulfate in supernatant of MLMECs or HUVECs-treated with Hep III for 2 h. **(C)** HUVECs were treated with 15 mU/mL Hep III for 2 h. Cells in the Hep III+SDX group were treated with 30 LSU/mL sulodexide for 2 h before Hep III treatment. Cells in the SDX group were treated with sulodexide for 2 h, and then with PBS in the same volume. Representative images of immunofluorescence of SDC1 on HUVECs, scale bar = 200 μm. **(D)** ELISA to detect the levels of SDC1 in supernatant of HUVECs-treated with Hep III for 2 h. **(E)** MLMECs were treated with 15 mU/mL Hep III for 2 h, 4 h, or 8 h. Cells in Hep III+SDX group were treated with 30 LSU/mL sulodexide for 2 h before. Cells in the SDX group were treated with sulodexide for 2 h, and then with PBS in the same volume. *SDC1* mRNA expression was measured by RT-qPCR. Data are presented as the mean ± SEM. **p* < 0.05; ***p* < 0.01.

Supplementary Figure 3 Sulodexide improved endothelial permeability resulting from glycocalyx shedding-induced ZO-1 disruption. **(A)** HUVECs were treated with 15 mU/mL Hep III for 2 h, and/or 30 LSU/mL SDX for 2 h, respectively. Relative fluorescence value of FD40 that passed through the inserts was assayed. **(B)** The levels of Zonula occludens-1 (ZO-1) and VE-cadherin were quantified by western blot for 8 h. **(C)** Statistical analysis of the levels of VE-cadherin for 2 h, 4 h, or 8 h. Data are presented as the mean ± SEM; ***p* < 0.01, ****p* < 0.001.

Supplementary Figure 4 Heparinase III induced the activation of NF-κB signaling. **(A, B)** MLMECs **(A)**/HUVECs **(B)** were treated with 15 mU/mL Hep III for 0 min, 15 min, 30 min, 60 min, 120 min. The levels of NF-κB/p-p65 and NF-κB/p65 were quantified by western blot. Data are presented as the mean ± SEM; **p* < 0.05.

Supplementary Figure 5 Inhibition of SDC1 shedding by Sulodexide improved lung injury and in CLP-induced sepsis mice. Sulodexide (40 mg/kg) was administered to mice for 2 h and then CLP was administrated for 24 h. **(A)** Representative lung tissue sections stained with HE at ×100 **(D)** in CLP-induced sepsis model. **(B)** Lung tissue W/D weight ratio in CLP-induced sepsis model. **(C)** Representative immunofluorescence images of SDC1 in lungs, scale bar = 200 μm. Data are expressed as means ± SEM; **p* < 0.05.
