# Supplementary material for: Sulodexide improves vascular permeability via glycocalyx remodelling in endothelial cells during sepsis
Source: Front Immunol. 2023 Aug 8;14:1172892. doi: 10.3389/fimmu.2023.1172892 (PMC10444196; doi:10.3389/fimmu.2023.1172892)
Supplement: Supplementary file 1 [file Table_1.docx]

**Table 1. Primers for RT-qPCR**

|  | Forward | Reverse |
| --- | --- | --- |
| SDC1 | 5’-GCCTACCAGAAACCCACCAA-3’ | 5’-CACAGAAGAGGCAAGTGGGA-3’ |
| β-actin | 5′-GTGCCCATCTACGAGGGTTA-3′ | 5′-TCTCAGCTGTGGTGGTGAAG-3 |
