# Supplementary material for: Sulodexide improves vascular permeability via glycocalyx remodelling in endothelial cells during sepsis
Source: Front Immunol. 2023 Aug 8;14:1172892. doi: 10.3389/fimmu.2023.1172892 (PMC10444196; doi:10.3389/fimmu.2023.1172892)
Supplement: Supplementary file 2 [file Table_2.docx]

|  | Total (n=28) | Survival (n=19) | Death (n= 9) | P |
| --- | --- | --- | --- | --- |
| Male, n (%) | 20 | 15 (78.9) | 5 (50.0) | 0.209 |
| Age, months | 9.2 (6.1, 31.2) | 10.5 (6.0, 43.0) | 8.0 (6.2, 9.7) | 0.176 |
| Infection site |  |  |  |  |
| Lung, n (%) | 20 (71.4) | 13 (68.4) | 7 (77.8) | 0.604 |
| Blood, n (%) | 5 (17.9) | 4 (21.0) | 1 (11.1) | 0.507 |
| CNS, n (%) | 2 (7.1) | 1 (5.3) | 1 (11.1) | 0.587 |
| Abdomen, n (%) | 1 (3.6) | 1 (5.3) | 0 (0) | 1.000 |

**Table 2. Clinical characteristics of 28 children with septic shock**

CNS, central nervous system

Differences among categorical variables were evaluated using the χ2 test. The Mann-Whitney U test was used to compare continuous data between children who survived and those who died.
