# Supplementary figures and images for: Sulodexide improves vascular permeability via glycocalyx remodelling in endothelial cells during sepsis
Source: Front Immunol. 2023 Aug 8;14:1172892. doi: 10.3389/fimmu.2023.1172892 (PMC10444196; doi:10.3389/fimmu.2023.1172892)

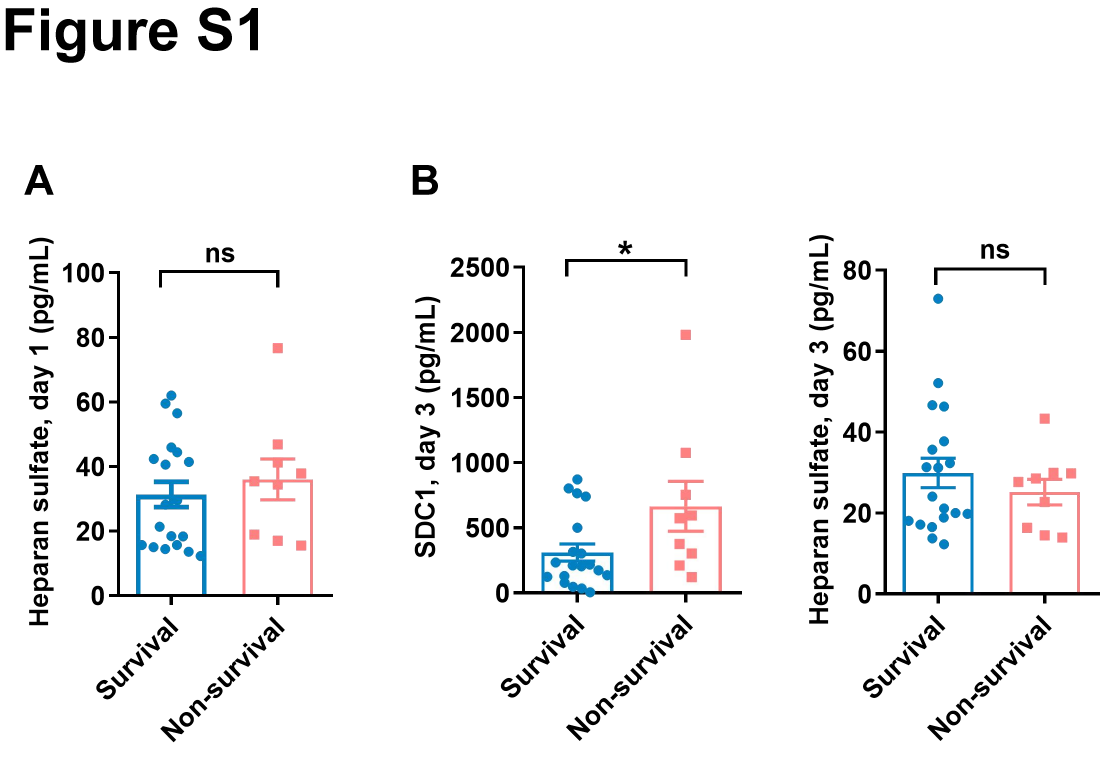

Supplement: Supplementary Figure 1 — Glycocalyx as biomarkers to predict the prognosis of children with septic shock. (A) ELISA to detect the levels of heparan sulfate in plasma from children with sepsis on day 1. (B) Levels of SDC1 and heparan sulfate in plasma from septic children on day 3. Bars and error bars represent the mean ± SEM; *p < 0.05; ns, no significance. [file Image_1.tif]

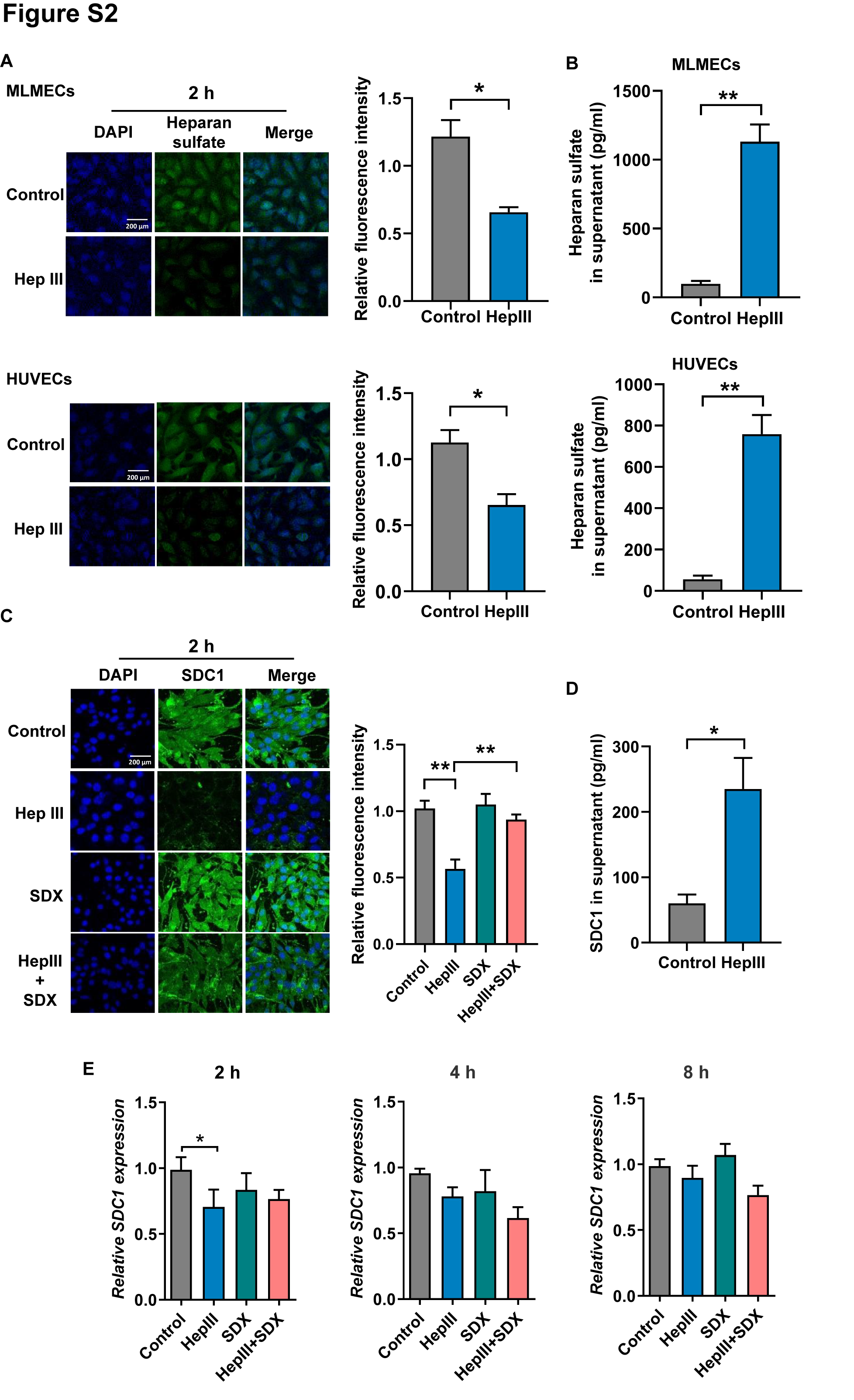

Supplement: Supplementary Figure 2 — Sulodexide decreased heparinase III-induced shedding of SDC1 in MLMECs/HUVECs. (A) MLMECs or HUVECs were treated with 15 mU/mL Hep III for 2 h. Representative images of immunofluorescence of heparan sulfate, scale bar = 200 μm. (B) ELISA to detect the levels of heparan sulfate in supernatant of MLMECs or HUVECs-treated with Hep III for 2 h. (C) HUVECs were treated with 15 mU/mL Hep III for 2 h. Cells in the Hep III+SDX group were treated with 30 LSU/mL sulodexide for 2 h before Hep III treatment. Cells in the SDX group were treated with sulodexide for 2 h, and then with PBS in the same volume. Representative images of immunofluorescence of SDC1 on HUVECs, scale bar = 200 μm. (D) ELISA to detect the levels of SDC1 in supernatant of HUVECs-treated with Hep III for 2 h. (E) MLMECs were treated with 15 mU/mL Hep III for 2 h, 4 h, or 8 h. Cells in Hep III+SDX group were treated with 30 LSU/mL sulodexide for 2 h before. Cells in the SDX group were treated with sulodexide for 2 h, and then with PBS in the same volume. SDC1 mRNA expression was measured by RT-qPCR. Data are presented as the mean ± SEM. *p < 0.05; **p < 0.01. [file Image_2.tif]

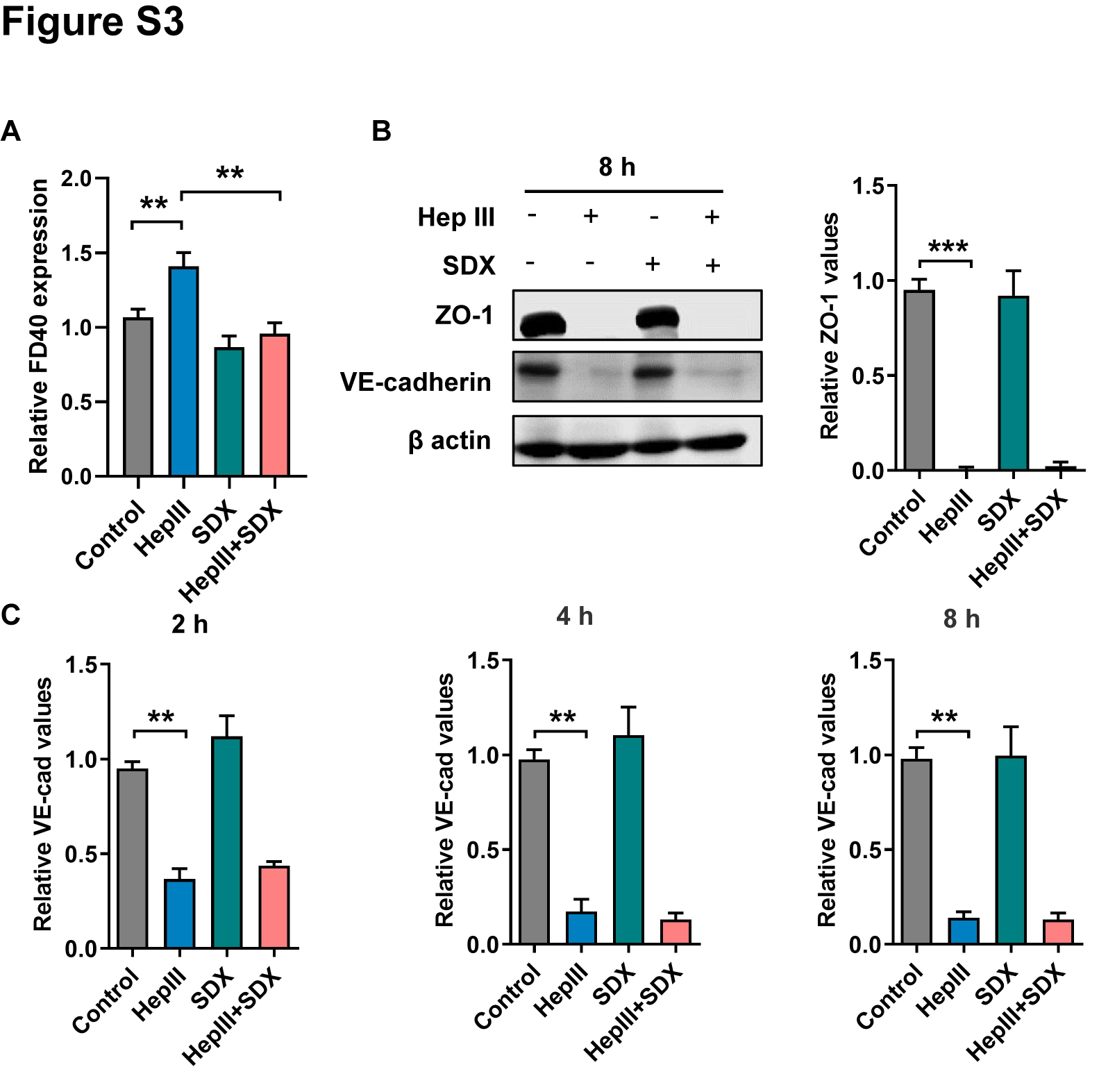

Supplement: Supplementary Figure 3 — Sulodexide improved endothelial permeability resulting from glycocalyx shedding-induced ZO-1 disruption. (A) HUVECs were treated with 15 mU/mL Hep III for 2 h, and/or 30 LSU/mL SDX for 2 h, respectively. Relative fluorescence value of FD40 that passed through the inserts was assayed. (B) The levels of Zonula occludens-1 (ZO-1) and VE-cadherin were quantified by western blot for 8 h. (C) Statistical analysis of the levels of VE-cadherin for 2 h, 4 h, or 8 h. Data are presented as the mean ± SEM; **p < 0.01, ***p < 0.001. [file Image_3.tif]

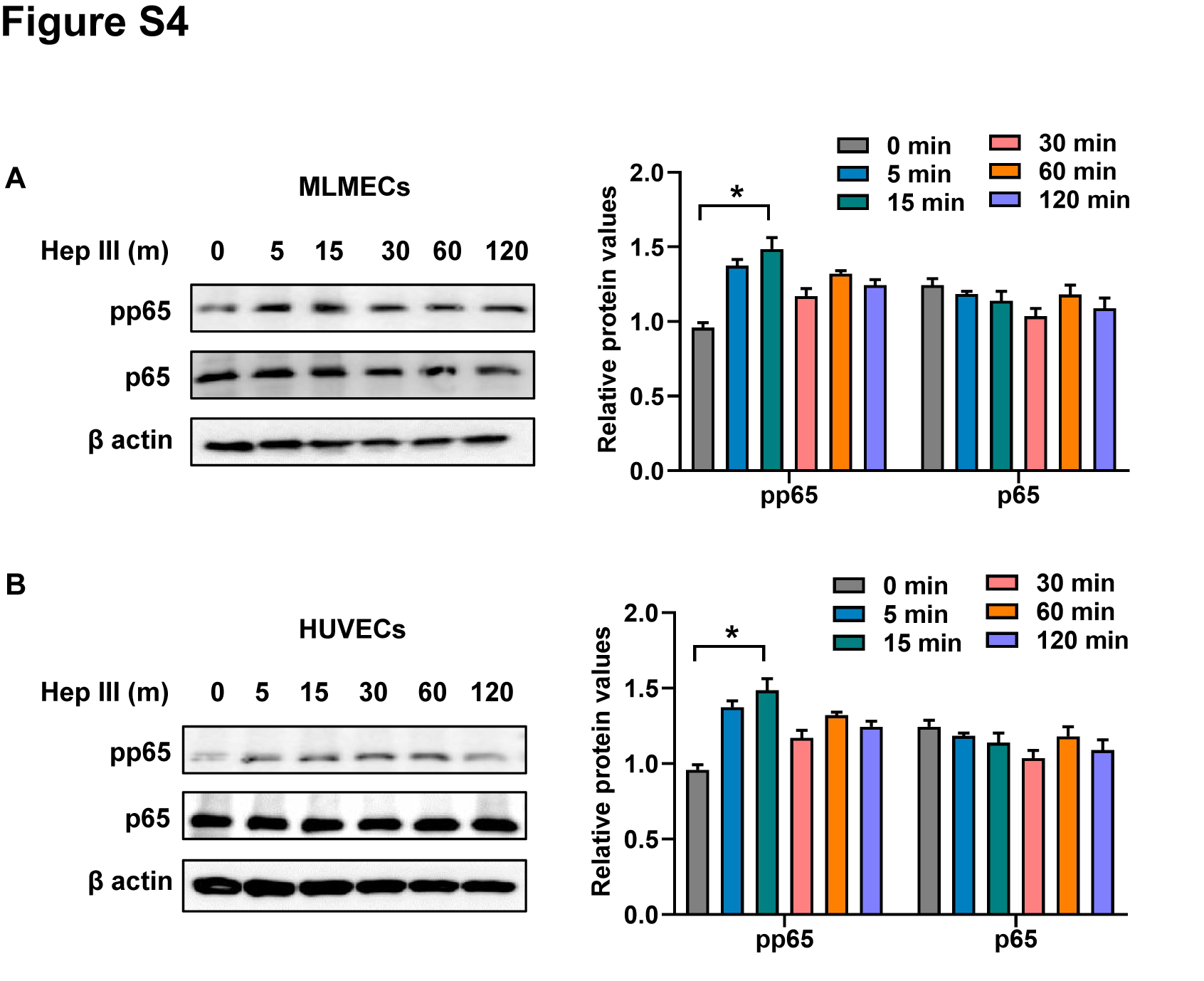

Supplement: Supplementary Figure 4 — Heparinase III induced the activation of NF-κB signaling. (A, B) MLMECs (A)/HUVECs (B) were treated with 15 mU/mL Hep III for 0 min, 15 min, 30 min, 60 min, 120 min. The levels of NF-κB/p-p65 and NF-κB/p65 were quantified by western blot. Data are presented as the mean ± SEM; *p < 0.05. [file Image_4.tif]

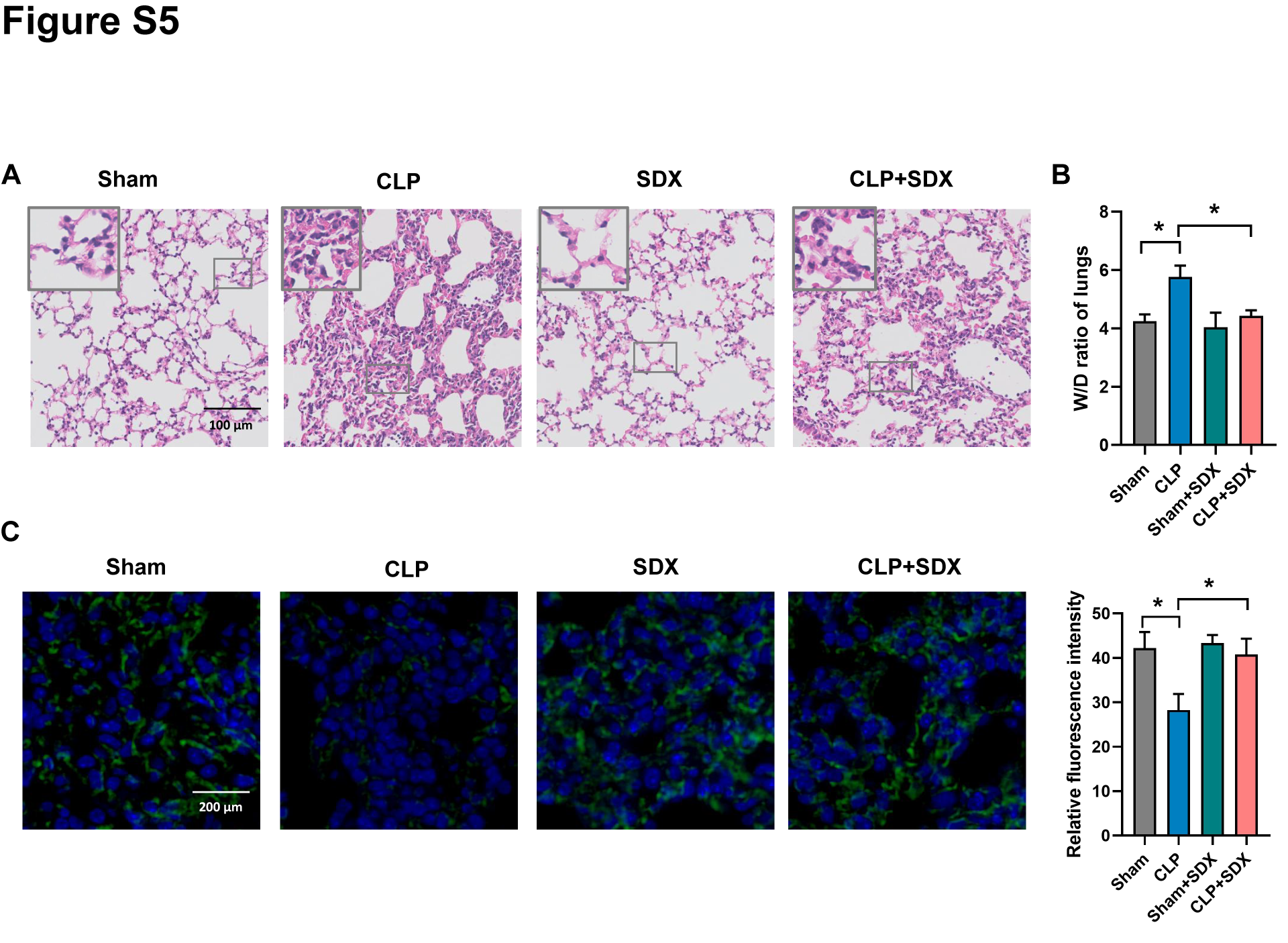

Supplement: Supplementary Figure 5 — Inhibition of SDC1 shedding by Sulodexide improved lung injury and in CLP-induced sepsis mice. Sulodexide (40 mg/kg) was administered to mice for 2 h and then CLP was administrated for 24 h. (A) Representative lung tissue sections stained with HE at ×100 (D) in CLP-induced sepsis model. (B) Lung tissue W/D weight ratio in CLP-induced sepsis model. (C) Representative immunofluorescence images of SDC1 in lungs, scale bar = 200 μm. Data are expressed as means ± SEM; *p < 0.05. [file Image_5.tif]
